# Site-specific functionalization of proteins and their applications to therapeutic antibodies

**DOI:** 10.5936/csbj.201402001

**Published:** 2014-02-14

**Authors:** Remko van Vught, Roland J Pieters, Eefjan Breukink

**Affiliations:** a Department of Membrane Biochemistry and Biophysics, Institute of Biomembranes, Utrecht University, Padualaan 8, 3584CH Utrecht, The Netherlands; b Department of Medicinal Chemistry and Chemical Biology. Utrecht Institute for Pharmaceutical Sciences, Utrecht University, P.O. Box 80082, 3508 TB Utrecht, The Netherlands

**Keywords:** protein labeling, site-specific modifications, antibodies, bioconjugation, bioorthogonal chemistry

## Abstract

Protein modifications are often required to study structure and function relationships. Instead of the random labeling of lysine residues, methods have been developed to (sequence) specific label proteins. Next to chemical modifications, tools to integrate new chemical groups for bioorthogonal reactions have been applied. Alternatively, proteins can also be selectively modified by enzymes. Herein we review the methods available for site-specific modification of proteins and their applications for therapeutic antibodies.

## Introduction

Proteins are the working horses of a living cell. Within and around cells they perform a magnificently diverse set of functions. Besides providing structure and stability, proteins are involved in cell signaling, catalyzing reactions, storage and transport, and are therefore extensively studied. Over the years, tools have become available for researchers to reveal structure and function relationships, as well as localization and their interactions with other proteins.

A relatively new tool is based on novel and specific chemistry. By modifying existing amino acids or introducing unnatural amino acids, proteins can be manipulated at the single amino acid level. Several methods involving the site-specific modification of proteins have been reported in the last decade. This allows the spatial and temporal control of proteins *in vivo*, as well as single molecule tracking. Modifications are introduced during protein translation, as post translational modification or chemically, after protein isolation.

Besides their usefulness for *in vitro/vivo* research, site-specific modifications are also interesting for therapeutic applications. Pharmaceutical companies have been refocusing their pipeline towards biological medicines (mainly monoclonal antibodies) because of the high specificity and safety. The ‘naked’ monoclonal antibodies have shown to be very effective in blocking receptors. A next generation of biological medicines are the antibody drug conjugates (ADCs), which efficiently deliver the payload to the target limiting the off target effects. Interestingly, site-specific modifications have also been applied to improve the properties of these therapeutic proteins.

Here, we review the tools for site-specific modification of proteins, followed by their applications in the development of therapeutic antibodies.

## Chemical modifications of proteins

The oldest and most straightforward method for labeling proteins is via the primary amino groups on lysine residues and at the N-terminus. In general, multiple accessible lysines and thus reactive amines are present on the protein surface, resulting in efficient labeling but inevitably leading to heterogeneous mixtures. Whether this method is applicable depends on the properties of the protein and the application. In the case of monoclonal antibodies, random labeling with fluorescent molecules hardly affects the antigen binding since many primary amines are present and only a small fraction may be important for this interaction. Smaller proteins such as antibody fragments are more likely to suffer from random conjugation due to a reduced number of lysines and the lack of an Fc region. There have been attempts to make this modification more specific by using preferential N-terminal labeling [1] or kinetically controlled lysine labeling [2]. Generally those methods suffer from low yields or require complex steps including the recycling of the original protein. Besides labeling the amino groups, similar obstacles exist for conjugation via carboxyl groups [3] and will therefore not be discussed in detail.

**Scheme 1:**
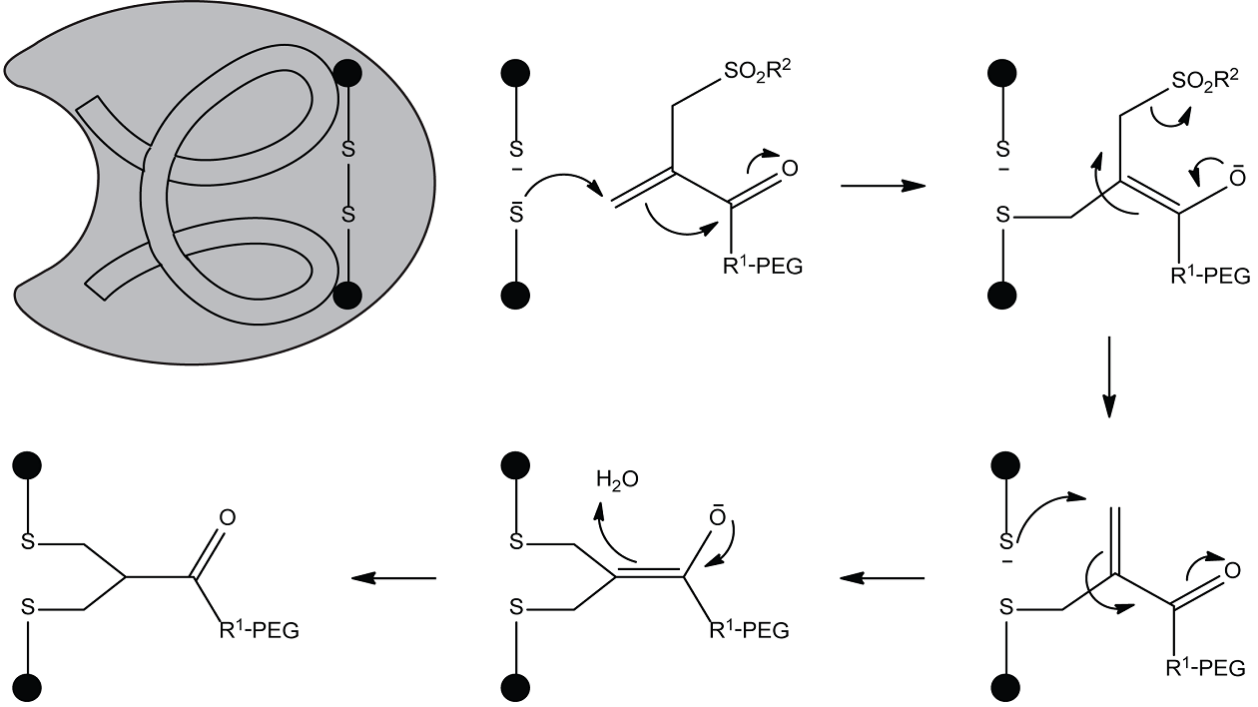
Double alkylation of proteins by PEG monosulfone-enone.

More selective is the labeling of proteins via sulfhydryl groups (also known as thiols). In proteins, most of the thiols are present in covalently linked pairs as disulfide bonds. The introduction of a cysteine by site-directed mutagenesis can be used for selective conjugation. Coupling reactions of maleimide groups with thiols have a high specificity over amines due to the lower pKa of the SH group (>1000 fold selectivity at pH 7.0) [4]. Therefore, cysteines are most commonly used for the site-selective modifications of proteins, though in some situations it is not feasible. One major drawback of introducing an extra cysteine is protein misfolding due to non-native disulfide bridge formation. In addition, thiol maleimide adducts have been reported to have limited stability *in vivo* [5]. Reactive thiols in albumin, free cysteine or glutathione can exchange with the existing thiol maleimide complex. Interestingly, hydrolysis of the succinimide ring prevented this exchange reaction [5]. Whether other alkylation reactions (with iodo/bromoacetamide analogs) also suffer from limited stability *in vivo* needs to be determined. Alternatively, an elegant double alkylation method by reducing disulfide bridges on the protein surface and subsequent conjugation with a PEG monosulfone-enone reagent was stable in human serum for over 30 hours and did not affect the protein stability (Scheme 1) [6].

Next to direct protein modification via alkylation, a reduced cysteine can be first converted to dehydroalanine. Subsequent nucleophilic addition by thiol modified biomolecules label the target protein via a thioether bond. This method is a straightforward route to natural occuring cysteine modifications including phosphor [7], farnesyl [8] and N-acetylhexosamine cysteine [9], and to structural mimics of post-translational modifications, but generates epimeric products due to loss of the stereocenter in the first step. Recently, several strategies for the conversion of cysteine to dehydroalanine have been evaluated [10].

Over the years, several site-specific chemical modifications methods have been reported for the N-terminal amino acids. N-terminal serine and threonine residues can selectively be oxidized by sodium periodate to form an aldehyde [11], followed by oxime ligation [12]. Besides oxime ligation, the oxidized serine was recently also used for the one step N-terminal dual protein functionalization using strain promoted alkyne–nitrone cycloaddition [13].

Proteins with N-terminal cysteines have been successfully used for reactions with thioesters [14] and applied for fusion proteins through native chemical ligation [15], which will be described in more detail later on.

More elegant methods are independent of the N-terminal amino acid. These approaches exploit the unique chemical properties of the N-terminus including the low pKa of the α-amino group of the N-terminus (8.9) compared to the pKa of the lysine e-amino group (10.5). Kinetically controlled lysine labeling is performed in small steps, using multiple additions of the label and allowing the most reactive amino group to be preferentially labeled [2].

Other methods are based on the introduction of unique reactive groups. The diazotransfer reagent imidazole-1-sulfonyl azide was shown to specifically convert the N-terminal amino group into an azide group [16]. The N-terminus can also be converted into a ketone or aldehyde group by a transamination reaction [17]. Peptide library screening identified residues with high yields (A, G, D, E, N,), other amino acids were either not/less reactive or were prone to side reactions [18]. In more recent work the transamination reaction was demonstrated for labeling of a monoclonal antibody [19]. Alternatively, N-terminal modification based on ketenes was applied to introduce an alkyne in peptides and proteins [20]. This reaction is highly specific for most N-terminal amino acids but yields range from 9 to 94%.

Although these methods are generally straightforward for peptides, applications for proteins predominantly depend on the solvent accessibility of the N-terminus. Moreover, small modifications limit the usefulness of reactions with low yields due to difficulties in separating the modified from the unmodified proteins.

## Metabolic modifications

**Figure 1:**
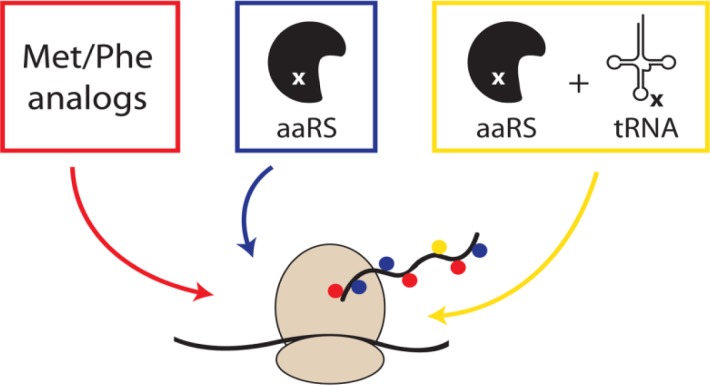
Metabolic labeling of proteins. The global replacement of natural amino acids by non-canonical analogs (red). Increased specificity for unnatural amino acids by manipulating the biosynthetic machinery (blue). Site-specific incorporation of unnatural amino acids by an orthogonal aminoacyl-tRNA synthetase/tRNA pair (yellow).

Metabolic labeling of proteins involves the replacement of one or more canonical amino acids by non-canonical analogs. The first observations by Munier and Cohem showed the incorporation of phenylalanine and methionine analogs in bacterial proteins (red scheme in Figure 1) [21]. Since then, many analogs have been synthesized and tested in auxotrophic bacterial hosts for incorporation at the expense of canonical amino acids [22]. The strict biological machinery accepts only minor modifications such as alkenes [23], alkynes [24] and azides [24] as amino acid side chains. The latter being of particular interest due to their compatibility with the Staudinger ligation and (copper-free) click chemistry [25].

The occurrence of multiple phenylalanine or methionine residues in proteins results in protein mixtures upon conjugation. Recently though, only one out of five azidohomoalanines of native CalB was shown to be surface accessible and reactive for functionalization [26].

Instead of designing amino acid analogs to be accepted by the biological machinery, advances have been made to manipulate the biosynthetic apparatus itself. Mutations in phenylalanyl-tRNA synthetase (PheRS) caused either an increase or decrease of the binding pocket size, and thus a change in the specificity towards phenylalanine analogs (blue scheme in Figure 1) [27]. The unnatural amino acid *p*-chlorophenylalanine could be incorporated into *Photinus pyralis* luciferase by expression of the mutant PheRS (A294G) in *E. coli*, replacing all phenylalanines [28]. More recently, the same has been demonstrated for non-canonical analogs using mutations in LeuRS [29], PheRS [30] and ValRS [31].

Both previous methods relied on the global replacement of canonical amino acid in proteins. The first site-specific modification of a single amino acid (based on editing the biological machinery) has been reported by Schultz [32] and Chamberlin [33] (yellow scheme in Figure 1). Non-canonical amino acids were incorporated upon suppression of the amber nonsense codon (TAG) by chemically acylated suppressor transfer RNA. In theory, also the two other stop codons could be targeted. However, the least used codon (TAG/amber codon) was selected to minimize the effect of translation read through on other proteins by suppression of the stop codon. This approach was first limited to *in vitro* production of proteins, or *in vivo* by microinjection into oocytes [34]. Later on, next to the gene of interest also the synthetase/tRNA pair was expressed *in vivo*. Efforts by Schultz and coworkers have improved this approach by applying selection schemes to reduce the interaction with the biological machinery [35, 36]. These include the selection with toxic genes bearing several amber codons to reduce the incorporation of canonical amino acids, as well as GFP expression in the presence of the unnatural amino acid to screen for the highest incorporation. In another example the whole biological machinery for the synthesis and incorporation of the 21^st^ amino acid was introduced in *E. coli* [37]. Moreover, the methodology has also been transferred to yeast and to mammalian cell lines by stable transfection.

Next to the suppression of non-sense codons, frame shift suppression has been used for the site-specific introduction of non-canonical amino acids [38]. This allows for not the triplet-base codon but codons containing 4 or 5 bases to be recognized. The usage of frameshift codons is complicated by competition by the endogenous triplet recognizing tRNA, resulting in a -1 frameshift and a premature termination. Alternatively, the frameshift suppressor tRNA could also recognize endogenous codons (3 + 1), causing a +1 frameshift and a premature termination as well. The selection of four-base codons based on genetic occurrence frequency allowed the incorporation of multiple unnatural amino acids *in vivo* [39].

Efforts over the years have allowed over 70 novel amino acids to be genetically incorporated via this approach, including photocrosslinkers, photocaged groups and fluorescent labels [37].

## Post translational modifications

After translation, almost all proteins require post-translational modifications (PTMs) before becoming mature. The oxidation of cysteines is a common PTM and is important for protein folding and stability. Other PTMs increase the functional diversity of proteins by the modification of amino acids including phosphorylation, glycosylation, ubiquitination, nitrosylation, methylation, acetylation and proline cis-trans isomerization [40]. Site-specific enzymatic PTMs are of particular interest since they can be used to manipulate and/or study proteins.

### Membrane associated modifications

Lipid modifications change the subcellular localization of proteins and can affect protein function [41]. Farnesyl- and N-myristoyltransferases recognize a consensus motif (CAAX and GXXXS/T) on proteins and subsequently conjugate farnesyl and myristoyl groups, respectively [42, 43]. Azide functionalized analogs of those groups have been used to label and study proteins [44, 45]. Although these transferases are sequence specific, the subcellular localization limits the applications for other proteins.

Another more random modification is introduced by transglutaminases (TGs), which cross-link proteins with isopeptide bonds between Lys and Gln residues [46]. TGs are involved in cell adhesion, stabilization of the extracellular matrix, apoptosis and wound healing. Importantly, multilayered epithelium, stabilized by these cross-links, protects the organism from the environment. The random crosslinking activity limits the possible applications of TGs. Cell surface proteins bearing a Q-tag (PNPQLPF, PKPQQFM, GQQQLG, and the recently identified RLQQP [47]) have been successfully labeled with biotin and fluorescein, though background labeling was observed [48].

### Formylglycine generating enzyme

In another approach the native formylglycine generating enzyme (FGE) is used to introduce formylglycine in both prokaryotes[49] and eukaryotes [50]. The aldehyde tagged protein can be readily functionalized with aminooxy- or hydrazide-functionalized biomolecules [50]. A drawback is the hydration of formyglycine in water to the diol-formylglycine, lowering the yield to around 85% [51].

### Self-modifications

Besides the modification of other proteins, some enzymes can be used for self-modification such as human O6-alkylguanine-DNA alkyl transferase (hAGT) [52], cutinase [53] and halo alkane dehalogenase [54]. Structural analogs of the natural substrates temper the biological function of hAGT and cultinase. A single mutation in halo alkane dehalogenase (His272Phe) traps the protein at an intermediate state and allows covalent attachment of chemical probes[54]. Fusion proteins bearing these domains can be selectively modified *in vitro* or *in vivo* [55–57]. Compared to other approaches, the large size of these domains (21-33 kDa) is considered as the major drawback. This can influence the function and/or localization of the protein of interest by the interaction with other biomolecules. Nevertheless, hAGT is commonly used for cell imaging studies because of the high labeling efficiency and of cell permeable probes [55, 58].

### Ligases

**Figure 2:**
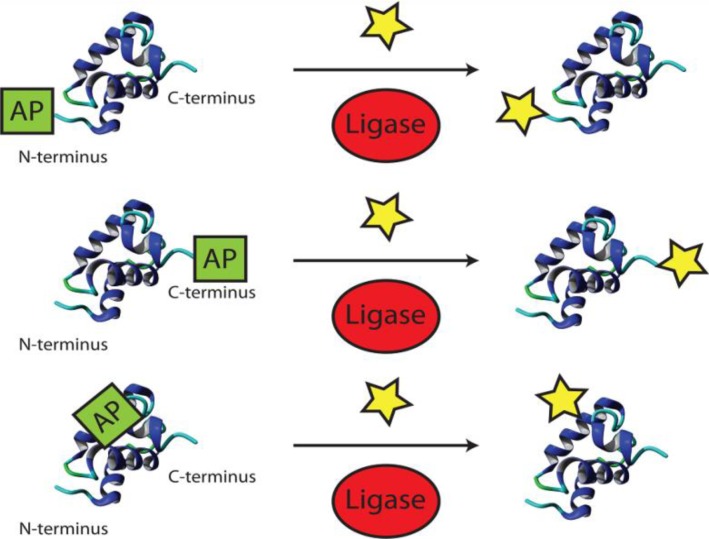
Protein modification by ligases at the N/C-terminus and in flexible loops.

A straightforward class of enzymes for modifying proteins after translation are the ligases (Figure 2). Ting and coworkers have been involved in exploiting several enzymes for site-specific modifications. First, biotin ligase (BirA) was shown to accept also a ketone isostere of biotin as a cofactor [59]. Ligation of this biotin analog to proteins bearing the 15-amino-acid acceptor peptide (AP) was demonstrated *in vitro* and *in vivo*, followed by subsequent ketone-hydrazine conjugation. Second, the microbial lipoic acid ligase (LplA) was used to specifically attach an alkyl azide onto proteins with an engineered LplA acceptor peptide (LAP) [60]. Although only 33% could be converted [61], cell surface labeling with cyclo-octyne probes was demonstrated [60]. Mutants of LplA were shown to be more efficient[61] (up to 89%) and also transfer fluorinated aryl azide [62] and 7-hydroxycoumarin [63] to LAP proteins for photocrosslinking and life cell imaging, respectively. More recently the portfolio of lipoic acid ligase was extended to ligate a trans-cyclooctene [64]. The Diels-Alder cycloaddition allows rapid labeling of inner and outer cellular proteins, though the yield is unknown.

### Transferases

Another set of post-translational modifications is performed by phosphopantetheinyl transferases (PPTases) [65]. PPTases are categorized into Sfp-like (B. subtilis) [65], AcpS-like (E. coli) [66] and FAS2-like (S. cerevisiae) [67] subfamilies and transfer a phosphopantetheinyl (P-pant) group through a phosphodiester bond onto peptidyl/acyl carrier protein (PCP/ACP) domains. These typically 80–120 residues long domains are present on nonribosomal peptide synthetases (NRPSs), polyketide synthases (PKSs), and fatty acid synthases (FASs)^65^.

Broad substrate specificity [68] and rapid conversion (>80% after 30 min) [69] was reported for Sfp-based labeling of proteins with phosphopantetheinylated analogs. In order to overcome possible size limitations, phage display screening identified several 11/12-residue peptide tags as replacement for the carrier domain, each allowing the labeling of N- or C-termini as well as flexible loops on target proteins [69, 70]. Interestingly, orthogonal fluorescent labeling of cell surface receptors was demonstrated by using Sfp and AcpS selective peptide tags [70].

### Transpeptidase

**Figure 3:**
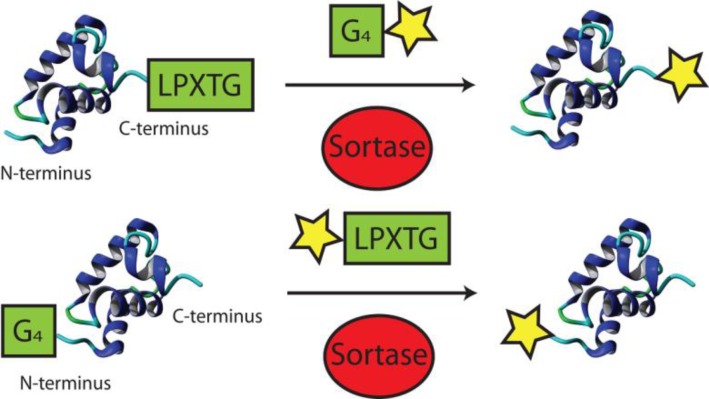
N- and C-terminal protein modification by sortases. Although sortase recognition sites have been engineered in flexible loops of proteins, the subsequent cleavage of the peptide backbone limits its therapeutic applications

Instead of exploring the chemical space in which biomolecules can be modified by functional groups and subsequently incorporated in proteins of interest, some general applicable enzymatic modifications preexist in nature. Sortases function as transpeptidase anchoring proteins to the bacterial cell wall [71]. Upon recognition of the sorting motif LPXTG (or LPXTA) a catalytic cysteine cleaves the peptide bond between residue T and G, yielding a thioacyl intermediate [72]. Instead of hydrolysing a peptide bond (as in the case of cysteine proteases), sortases accept a N-terminal (oligo)glycine as a nucleophile, creating a new peptide bond between the two molecules (Figure 3).

Sortases function at physiological conditions and have been used for protein labeling with various functionalities such as biotin, fluorophores, cross-linkers and multifunctional probes [73]. Target proteins are commonly labeled C-terminally with the LPXTG sorting motif, followed by a purification tag. Subsequent transpeptidation removes the purification tag and generates the labeled proteins in high yields. Interestingly, this approach has also been used to study the structure and function of a solvent-exposed loop within the ubiquitin C-terminal hydrolase 3 protein [74].

Besides introducing the sorting motif, proteins can also be equipped with a N-terminal (oligo)glycine for N-terminal conjugation. In this case, the sortase recognition element should be introduced onto the biomolecule. This approach has mostly been used for bacterial cell wall labeling with biotin, azide and fluorescent groups [73].

Alternatively, also both N- and C-terminal ligation has been demonstrated [75]. Selective labeling is achieved by using two sortases with different specificity (LPXTG & LPXTA), preventing the oligomerisation of proteins. Protein cyclisation occurs in cases where the N- and C-termini are in close proximity [76, 77]. This is of particular interest in therapeutic drug design due to the enhanced conformational stability and increased resistance to proteolytic cleavage [78].

The fusion of two proteins can be achieved in a similar fashion, with each protein bearing one of the tags. Although genetic fusion of proteins is much more straightforward, in some cases this is not feasible. For instance when protein folding is affected, protein yields drop or proteins come from different hosts. In a recent study, 10 pairs of protein domains were generated with yields between 40-85% [79].

The labeling of proteins by sortases has been optimized and well described. One of the major drawbacks is the high concentration of sortase required. The poor reaction kinetics have been improved 140-fold using directed evolution by increasing the affinities for the sorting motif LPXTG as well as the (oligo)glycine peptide [80].

### Protein splicing

Inteins, also known as protein introns, are protein domains expressed in frame of another protein [81]. Removal of the intein domain by self-excising, rejoins the two external host protein segments by formation of a native peptide bond, and restores the function of the host protein.

**Figure 4:**
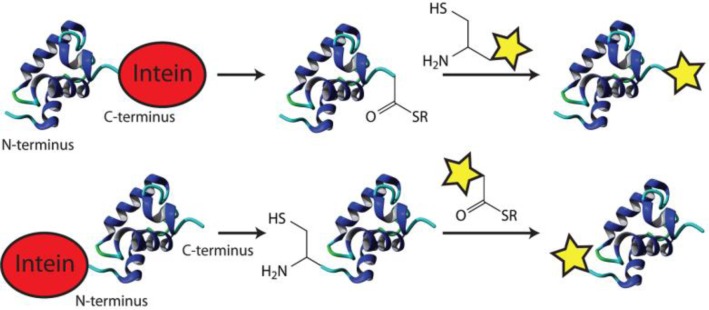
Intein-mediated conjugation of biomolecules.

This process can be exploited for the N/C-terminal ligation of biomolecules (Figure 4) [82]. The C-terminal labeling requires the formation of a thioester by nucleophilic attack of the intein N-terminal cysteine. The thiol can be exchanged in the presence of thiol reagents, resulting in cleavage of the intein. In a subsequent native chemical ligation reaction with a cysteine functionalized molecule, the thiol exchanges again followed by the generation of a peptide both by the S-N shift. The C-terminal intein mediated conjugation has been demonstrated for labeling with biotin [83], fluorophores [84] and lipids [85, 86]. Moreover, semi synthetic proteins were produced by the ligation of cysteine bearing peptides, known as expressed protein ligation [87].

The N-terminal labeling also requires the exchange of thiols for cleaving off the intein. Now the intein C-terminal asparagine breaks the peptide bond, freeing an N-terminal cysteine on the protein of interest. Labeling of N-terminal cysteine is performed in the same NCL reaction with a thioester modified biomolecule and has been used to immobilize proteins on microarrays [88] as well as for *in vivo* labeling [89].

Inteins can, similarly to sortases, also facilitate the cyclisation of proteins[82]. The commercialized IMPACT kit allows straightforward production and purification of proteins with an N- and/or C-terminal modification for site-specific functionalization [15, 90]. As the intein domain is coexpressed, no other proteins are required. However, reactions in complex mixtures are challenging since thioesters can be inactivated by reactions with amines and by hydrolysis [91].

### Glycosylation

The covalent attachment of carbohydrate chains (glycans) to proteins is the most prevalent and complex PTM, also known as glycosylation [92]. Glycans can be N-linked to proteins via the asparagine or arginine side-chain, or O-linked via the hydroxyl group mostly on serine, threonine and tyrosine, and also hydroxylysine, or hydroxyproline side-chains [93]. Although the majority of the glycoproteins are present on the exterior surface of cells, the O-GlcNAc modification has also been reported for proteins in the cytosol and nucleus [94].

Glycosylation is important for protein folding and stability, thereby affecting the circulation lifetime in blood (discussed later on) [95]. Interestingly, the PTM itself can be used for subsequent modification of glycoproteins via bioorthogonal chemistry. For instance, the metabolic labeling of glycans is achieved by feeding cells or organisms with modified glycan precursors [96]. Several azido sugars including N-azidoacetylmannosamine (ManNAz), N-azidoacetylgalactosamine (GalNAz), N-azidoacetylglucosamine (GlcNAz) and 6-azidofucose (6AzFuc) have been incorporated into glycoproteins by the glycan biosynthetic machinery in both *in vitro* and *in vivo* [97]. In addition, attempts have been made to enzymatically label glycoproteins. For example the permissive mutant β-1,4-galactosyltransferase (Gal-T1 (Y289L)) introduces azido galactosamine (GalNAz) onto O-GlcNAc–modified glycoproteins [98, 99]. The introduced azido group allows subsequent glycan-profiling and visualization of proteins of interest [98].

Although the glycan modification of glycoproteins expands the researcher's toolbox, the great structural complexity limits its applications today. Especially the glycan recognition by the immune system affects the usefulness for therapeutic proteins [100]. Attempts to overcome these problems involve exploring the production of therapeutic glycoproteins in different hosts and addressing (chemo) enzymatic methods to derive homogeneous glycosylation patterns.

## Applications for therapeutic antibodies

Traditionally, drugs have been small chemical entities based on natural and (semi)synthetic products [101]. Analogs of natural active compounds have been optimized for physicochemical and pharmacological properties allowing oral administration while maintaining therapeutic efficacy. The lack of specificity and/or the inability to block protein-protein interactions by these small chemical entities stimulated the development of protein based drugs [102]. Certain type of proteins such as hormones and antibodies naturally bear very high specificity for their target. Moreover, their natural appearance in the human body makes them reasonably safe as therapeutic compounds. Protein drugs, however, often suffer from other issues such as low stability, poor pharmacokinetics, limited efficacy and require a complex route of administration [103, 104]. Over the years, research groups and pharmaceutical companies have made various attempts to improve these parameters by modifying therapeutic proteins using some of the above mentioned methods [105]. The second part will review these modifications.

### Pharmacokinetics

*In vivo* responses of (protein) therapeutics are influenced by drug absorption, distribution, metabolism and excretion (ADME). Small sized proteins are predominantly cleared via glomerular filtration by the kidneys [106]. This results in a half-life of 2 hours for single-domain antibodies (15 kDa) [107] and 12-20 hours for Fab fragments (50 kDa) [108]. Proteins above the glomerular filtration cutoff (molecular weight >50 kDa and hydrodynamic radius >60 Å) are cleared by other pathways including proteolytic degradation, hepatic uptake and immune clearance[109]. Monoclonal antibodies, for instance, are 150 kDa and have long half-lives (7-23 days) by default [110].

The elimination of small proteins by the kidneys can thus be influenced by modifications affecting the size. The covalent attachment of water soluble polymers to proteins (such as polyethylene glycol; PEG) increases the hydrodynamic size and interestingly also reduces the immunogenicity by masking the protein from the immune system [111]. Several branched and non-branched PEG structures have been evaluated for the effect on renal clearance. Enhanced PK profiles for branched PEG conjugates have consistently been described for therapeutic proteins in the literature [112–114]. Recently for instance, single domain antibodies labeled with 2x20 kDa PEG were shown to be superior over 1x40 kDa and 4x10 kDa labeling without affecting the biological activity [114]. Biodistribution studies showed higher serum exposure of the antibody, though this was not the case for some tissues.

Although PEG is considered as the golden standard in drug delivery, antibody formation against PEG conjugates was reported in 1983 already [115]. Interestingly, preexisting antibodies against PEG were found in healthy donors of the PEG-asparaginase clinical trial [116]. And even, a more rapid blood clearance of PEG conjugates was observed in patients with existing anti-PEG antibodies [116, 117]. This may seriously affect the applications of PEG for drug delivery due to an expected reduced therapeutic efficacy in patients with antibodies against PEG.

These issues stimulated researchers to find alternative polymers including non-biodegradable poly(glycerol)s, poly(vinylpyrrolidone), poly(2-oxazoline)s, poly(N-(2-hydroxypropyl)methacrylamide), and biodegradable poly(amino acid)s. Promising results were reported for some alternatives, however the current understanding is very limited and requires additional (clinical) studies. A recent review by Knop et al. discusses PEG and potential alternatives in more detail [118].

Monoclonal antibodies on the other hand have already a long half-life. For therapeutic purposes, the IgG class has predominantly been used. Within the IgG class, the IgG subclasses 1-4 differ in the Fc region which affects effector functions such as phagocytic cell recruitment and complement activation through cellular IgG-Fc receptors, and in half-life by recycling via neonatal Fc receptor (FcRn) [119]. The strong effector effects and long half-life of subclass IgG1 are ideal for antibody based therapy in oncology [120]. Other treatments such as in Crohn's disease mainly depend on antigen neutralization. Here, the effector functions by the Fc region can give rise to side effects. This led to the development of a pegylated IgG1-Fab (Certolizumab pegol) next to the existing IgG1's infliximab and adalimumab, which misses the Fc region [121]. An additional benefit is the lack of active transport by FcRn across the human placenta and thus the antibody should be safe in pregnancy. Alternatively strategies to reduce/remove effector functions are IgG isotype switching to IgG2/4 and removal of glycosylation sites [122]. The latter can be achieved either by mutation of asparagine 297 in the CH2 domain (and additional glycosylation sites) or by expression of antibody (fragments) in prokaryotic hosts.

### Distribution

Biodistribution is another factor determining the efficacy of antibody therapy, and varies per Ig class due to differences in the Fc region. The distribution of IgG class monoclonal antibodies is mainly confined to blood and extracellular fluid. Pegylation of antibody fragments was reported to influence the biodistribution [123]. Since the therapeutic response of antibody therapy depends on the drug concentration at the target site, many studies have analyzed impaired distribution in tumors [124–126]. Compared to normal tissue, the interstitial hydrostatic pressure and missing lymphatic draining restricts movement of antibodies [126]. Consequently, invasion of monoclonal antibodies occurs predominantly via diffusion across pores in the capillary and is limited by the molecular size. In addition, the ‘binding site barrier’ limits deep penetration of antibodies into tumors[127, 128]. Diffusion and convection are restricted to the outer layer of cancer cells due to high affinity, rapid internalization and subsequent metabolism of antibodies [129].

Taken together, tumor penetration can be improved by targeting smaller sized antibodies including single-domain antibodies and Fab fragments. These antibody fragments lack an Fc region and therefore have a reduced half-live. Whether the improved tumor penetration is sufficient to compensate for the shorter circulation time needs to be evaluated.

### Clearance

The molecular weight of monoclonal antibodies is beyond the filtration cutoff of the kidneys. Still, the half-life between IgG subclasses ranges from 7 to 23 days [110]. The interaction between the Fc region and the FcRn receptor, has been suggested as one of the determining factors. Where normally the uptake of proteins by vascular endothelial cells would result in degradation in lysosomes, antibodies are recycled back into the circulation as a consequence of their interaction with the FcRn receptor [130]. Validation in FcRn knockout mice showed a 10-15x higher IgG elimination while other classes were not affected [131–133]. In addition, engineered tighter binding to the FcRn resulted in a 2-fold increase in the half-life in monkeys [134]. Other factors determining the clearance rate of antibodies are immunogenicity, proteolysis and glycosylation [135]. Interestingly, the production of hyperglycosylated antibodies fragments in engineered cell lines demonstrated longer half-lives as well as reduced proteolysis [109]. Alternatively, Fab fragments conjugated to PEG benefits, beside from the increased size, also from the reduced intracellular uptake and proteolytic degradation by masking sensitive sites [114].

### Antibody drug conjugates (ADCs)

The next generation of biological medicines are the antibody drug conjugates (ADCs) [136]. Where ‘naked’ monoclonal antibodies rely on the recruitment of immune cells by the Fc region for its toxicity, ADCs bear a stable (or selective cleavable) linkage with a cytotoxic payload [137]. The release of the payload after absorption/internalization by the target cell minimizes exposure of healthy tissues [137]. More importantly, because of the cytotoxic payload, ADCs are more effective in the killing of cancer cells [138].

An interesting example is the monoclonal antibody trastuzumab (Herceptin), which targets Her2 over-expression on certain types of breast cancer [139]. Trastuzumab has been marketed since 1998 and predominantly inhibits tumor proliferation. Recently, ADC trastuzumab emtansine (Kadcyla) was approved by the FDA. The delivery of cytotoxic emtansine induces microtubule disruption, thereby making this construct more effective (54% longer median progression-free survival compared to trastuzumab plus docetaxel) [140, 141]. Between 0 and 8 emtansine molecules are randomly conjugated via the SMCC crosslinker onto lysine residues on trastuzumab (3.5 on average)[140]. Because of the on average 100 lysines per antibody, this results in a heterogeneous mixture. A 2/3-fold faster clearance of trastuzumab emtansine compared to the naked antibody has been attributed to deconjugation and proteolytic degradation of the ADC [142].

Better defined is the monoclonal antibody brentuximab vedotin (Adctris) for treating Hodgkin's lymphoma. Mild reduction by dithiothreitol (DTT) generates 8 thiol groups from four interchain disulfide bridges [143]. Monomethyl auristatin E (MMAE) is conjugated to 3-5 thiol groups (4 on average). Although labeling of brentuximab with 8 MMAE molecules has also been reported, the higher degree of labeling generally results in faster clearance/shorter half-life. Interestingly, the *in vivo* antitumor activity was comparable for ADCs bearing 4 or 8 MMAE molecules (at equal mg/kg/dose) [144].

Off target/side effects by ADCs can generally be explained by 3 situations: 1) The antibody is not specific enough causing accumulation in healthy tissue; 2) The cytotoxic agent is lost before it reaches the target cell; or 3) The heterogeneous population has altered specificities or pharmacokinetics. The drawbacks of producing monoclonal antibodies as heterogeneous product led to the site-specific incorporation of unnatural amino acids in monoclonal antibodies and antibody fragments [145–147]. A defined stoichiometry and stable linkage is expected to reduce the side effects of ADCs. In addition, optimal sites for conjugation can be selected to reduce the effect on the circulation time.

In order to demonstrate this, a noncleavable auristatin analog was conjugated to trastuzumab bearing the unnatural amino acid *p*-acetylphenylalanine (pAcF) [146]. The functionalized ADC was obtained in an overall yield of >95% and showed a similar clearance rate as the naked antibody.

Currently, the companies Allozyne, Ambrx and Sutro explore the incorporation of unnatural amino acids for its therapeutic applications. An overview of FDA approved ADCs is given in Table 1.

**Table 1:** Overview of FDA approved ADCs.

mAb	Drug	Drugs/Ab (average)	Chemistry	Reducable	Trigger	Status (US)	Ref
Gemtuzumab ozogamicin	Calicheamicin	4-6 (5*)	Lysine	yes	pH	approved (2000) & withdrawn (2010)	[148]
Brentuximab vedotin	Monomethyl auristatin E (MMAE)	3-5 (4)	Cysteine	no	protease	approved (2011)	[143]
Trastuzumab emtansine	Mertansine (DM1)	0-8 (3.5)	Lysine	no	none	approved (2013)	[140]

* For only 50% of the antibodies

### Bispecific antibodies

Next to ADCs, also the recruitment of T-cells by bispecific antibodies is effective for the treatment of cancer. Bispecific antibodies recognize tumor specific antigens and T-cells at the same time [149]. In one strategy, random crosslinking has been applied to conjugate two antibodies together via hetero-bifunctional crosslinkers such as SPDP (succinimidyl-3(2-pyridylthiol)propionate) [150–152] and SMCC (Succinimidyl-4-(N-maleimidomethyl)cyclohexane-1-carboxylate) [153]. Monoclonal antibodies however bear two fragment antigen-binding sites (Fab fragment) both recognizing the same antigen. Selective reduction of disulfide bonds and subsequent oxidation was used to acquire monovalent bispecific antibody fragments (one Fab for each antigen) [154].

More recently, uniform bispecific antibodies were generated by first expressing two sets of half-antibodies which were unable to dimerize [155]. The bispecific antibodies were spontaneously formed by mixing the reduced half-antibodies under oxidizing conditions. In contrast, the only FDA approved bispecific antibody therapy (Catumaxomab) is directly produced in hybrid mouse/rat quadroma cell lines [156]. Due to the homology in the hinge region between mouse and rat antibodies, the 30-49% yield almost reached the statistical limit of 50% (m/m, **m/r**, **r/m** and r/r) [157]. Besides binding tumor cells via the EpCAM antigen and T-cells via the CD3 receptor, the intact Fc region of Catumaxomab recruits accessory cells to enhance the immune response against the tumor [158].

Next to the generation of full monoclonal antibodies, two Fab fragments bearing genetically encoded unnatural amino acids were conjugated to form an anti-HER2/anti-CD3 bispecific antibody[145]. The confined sites and defined chemistry allowed homogeneous products in a two-step process. Although effective tumor killing was observed *in vitro*, the efficacy *in vivo* still needs to be determined.

### Nanoparticles

Based on antibody complexes in nature, immune complexes have emerged for the neutralization of antigens. Binding to tumor specific antigens blocks signaling cascades as well as causes down-regulation of the receptor [159]. Diverse set of scaffolds including avidin [160], gold [161], liposomes [162] and polymersomes [163–166] have been decorated with antibodies or antibody fragments. Recently, DNA scaffolds decorated with single-domain antibodies were demonstrated to allow various structures such as dimers and tetramers [147].

Besides immune complexes binding to antigens, the subsequent internalization has drawn attention for the delivery of drugs. Compared to current ADCs, nanocapsules facilitate the delivery of high drug concentrations by active (antibody binding) and passive targeting (EPR effect; beyond the scope of this review, see ref [167]). In order to remain in the blood circulation, nanoparticles need to meet several criteria including confined size, shape and chemical properties [168]. Antibody fragments are often used for the targeting of nanocapsules because no Fc region and subsequent signaling cascade is required. Since none of these nanomedicines have been FDA approved, an overview of decorated nanoparticles in clinical trials is given in Table 2.

**Table 2:** Antibody decorated nanoparticles in clinical trials.

Name	Particle	Drug	Chemistry	Target	Antibody	Phase	Ref
Erbitux^®^EDVsPAC	Bacterially-derived minicell	Paclitaxel	-	EGFR	mAb	II	[169]
SGT-53	Liposome	p53 gene	NA	Transferrin	scFv	Ib/II	[170]
MM-302	Liposome	Doxorubicin	NA	HER2	Fab	I	[171]
Lipovaxin-MM	Liposome	Melanoma antigens and IFNγ	-	DC-SIGN	sdAb	I	NA
SGT-94	Liposome	RB94 gene	NA	Transferrin	scFc	I	NA
C225-ILS-DOX	Liposome	Doxorubicin	Cysteine	EGFR	Fab (cetuximab)	I	[172]
MCC-465	Liposome	Doxorubicin	Lysine	Unknown	Fab_2_	I*	[173]

* Clinical trial is performed in 2004, current status is not available

## Summary and Outlook

Site-specific modification of proteins has emerged as powerful tool to study proteins at the single amino acid level. Currently, the field is expanding towards applications for therapeutic proteins. Several studies have demonstrated the usefulness of unnatural amino acids in antibody drug conjugates. The time-consuming drug development and approval process has delayed the integration of these methods for therapeutic antibodies, but this can be expected in the near future.

In contrast to therapy, the approval process for diagnostic antibodies is shorter. The functionalization methods described in this review would be ideal to label antibodies with diagnostic tracers (radioactive, fluorescent or contrast agents), but will be even more important for the successful development of theranostics, a one-molecule combination of diagnosis and therapy.
